# ^18^F-FDG PET/CT based spleen to liver ratio associates with clinical outcome to ipilimumab in patients with metastatic melanoma

**DOI:** 10.1186/s40644-020-00313-2

**Published:** 2020-05-14

**Authors:** Annie Wong, Jason Callahan, Marleen Keyaerts, Bart Neyns, Johanna Mangana, Susanne Aberle, Alan Herschtal, Sonia Fullerton, Donna Milne, Amir Iravani, Grant A. McArthur, Rodney J. Hicks

**Affiliations:** 1 Research Division, Peter MacCallum Cancer Centre, 305 Grattan St, Melbourne, VIC 3000 Australia; 2 Sir Peter MacCallum Department of Oncology, University of Melbourne, Melbourne, VIC 3010 Australia; 3 Department of Cancer Imaging, Peter MacCallum Cancer Centre, 305 Grattan St, Melbourne, VIC 3000 Australia; 4 Nuclear Medicine Department, UZ Brussel, Laarbeeklaan 101, 1090 Brussels, Belgium; 5 In Vivo Cellular and Molecular Imaging Laboratory, Vrije Universiteit Brussel, Laarbeeklaan 103, 1090 Brussels, Belgium; 6 Department of Medical Oncology, UZ Brussel, Laarbeeklaan 101, 1090 Brussels, Belgium; 7 Department of Dermatology, University Hospital Zurich, Zurich, Switzerland; 8 Department of Nuclear Medicine, University Hospital Zurich, Zurich, Switzerland; 9 Centre for Biostatistics and Clinical Trials, Peter MacCallum Cancer Centre, 305 Grattan St, Melbourne, VIC 3000 Australia; 10 Department of Palliative Care, Peter MacCallum Cancer Centre, Melbourne, Australia; 11 Department of Cancer Experiences Research, Peter MacCallum Cancer Centre, Melbourne, Australia

**Keywords:** Melanoma, Immune checkpoint blockade, Biomarker, Positron emission tomography, Spleen to liver ratio

## Abstract

**Background:**

Immune checkpoint blockade such as ipilimumab and anti-PD1 monoclonal antibodies have significantly improved survival in advanced melanoma. Biomarkers are urgently needed as a majority of patients do not respond, despite treatment-related toxicities. We analysed pre-treatment ^18^F-fluorodeoxyglucose positron emission tomography/computerised tomography (FDG PET/CT) parameters to assess its correlation with patient outcome.

**Methods:**

This retrospective study evaluated pre-treatment FDG PET/CT scans in a discovery cohort of patients with advanced melanoma treated with ipilimumab or anti-PD1. Pre-treatment scans were assessed for maximum tumoral standardised uptake value (SUVmax), metabolic tumour volume (MTV) and spleen to liver ratio (SLR). Progression-free survival (PFS) and overall survival (OS) were characterised and modelled using univariable and multivariable analyses. Correlation of SLR and OS was validated in an independent cohort. Blood parameters and stored sera of patients from the discovery cohort was analysed to investigate biological correlates with SLR.

**Results:**

Of the 90 evaluable patients in the discovery cohort: 50 received ipilimumab monotherapy, 20 received anti-PD1 monotherapy, and 20 patients received ipilimumab followed by anti-PD1 upon disease progression. High SLR > 1.1 was associated with poor PFS (median 1 vs 3 months; HR 3.14, *p* = 0.008) for patients treated with ipilimumab. High SLR was associated with poor OS after ipilimumab (median 1 vs 21 months; HR 5.83, *p* = 0.0001); as well as poor OS after first line immunotherapy of either ipilimumab or anti-PD1 (median 1 vs 14 months; HR 3.92, *p* = 0.003). The association of high SLR and poor OS after ipilimumab was validated in an independent cohort of 110 patients (median 2.3 months versus 11.9 months, HR 3.74). SLR was associated with poor OS in a multi-variable model independent of stage, LDH, absolute lymphocyte count and MTV.

**Conclusions:**

Pre-treatment Spleen to liver ratio (SLR) > 1.1 was associated with poor outcome after ipilimumab in advanced melanoma. This parameter warrants prospective evaluation.

## Background

The advent of immune checkpoint blockade has resulted in unprecedented improvements in survival for patients with metastatic melanoma. However, response rates of approximately 10 and 40% respectively for anti-CTLA4 (ipilimumab) [1, 2] and anti-PD1 monoclonal antibodies [3–5], indicate that the majority of patients do not respond. In 2015, the combination ipilimumab and nivolumab demonstrated improved response rates and progression-free survival compared to single agent ipilimumab [6, 7]. However, this combination resulted in significantly higher treatment-related toxicity than monotherapy (55% vs 16–27% patients experienced common toxicity criteria for adverse events grade 3 and 4 toxicities respectively). Even then, 42% of patients still failed to achieve an objective response. Hence, biomarkers that can predict clinical outcomes are urgently needed.

There are numerous candidate predictive biomarkers including expression of tumoral PDL1 [8, 9] and tumoral mutational load [10], but these have not entered clinical practice due to their inability to clearly prospectively identify responders from non-responders. It is now recognised that a combination of different biomarkers may be needed to predict response given the complexity of the immune interaction with tumour [11]. Blank et al. suggested a ‘cancer immunogram’ wherein a combination of biomarkers such as tumoral mutational load, presence of T-cell checkpoints, soluble cytokines, metabolic factors and host immune factors should be considered. Here we explore how existing non-invasive functional imaging might provide a readily translatable source of novel biomarkers, given the current increase of ^18^F-fluorodeoxyglucose positron emission tomography/computerised tomography (FDG PET/CT) in the surveillance of high-risk melanoma [12] or at suspected relapse [13].

^18^F-fluoro-deoxy-glucose positron emission tomography (FDG PET) has long been the preferred functional imaging technique in melanoma in our facility [14]. Our identification of malignancy is largely qualitative based on a combination of PET and CT appearances [15] since the use of the semi-quantitative measure of uptake intensity termed the standardised uptake value (SUV) can have limitations with regards to cancer evaluation due to the large number of technical factors that can influence its measurement [16]. Nevertheless, the measurement of SUVmax has been shown to have some prognostic significance in a number of malignancies, including melanoma [17]. Metabolic tumour volume (MTV) is a measure of metabolically active disease and, reflecting the burden of disease, might be more likely to correlate with clinical outcome.

In addition to studying PET-derived parameters of the tumour, there may be merit in examining the patients’ tissues, such as the spleen or draining lymph nodes, as a way of characterising immune status. The SUV of normal organs, particularly the liver and spleen, generally have a rather narrow range [18, 19]. For example, the liver normally exhibits homogenous uptake of FDG (normal SUVmax of liver in men range from 2.3–5.0 and 2.3–3.8 in women) [20], unless there is obvious disease infiltration or sarcoidosis. The spleen is the largest lymphoid organ in the body, and normally has less FDG uptake compared to the liver (normal SUVmax of spleen in men range from 1.6–4.1 and 1.6–3.2 in women), with an expected Spleen to Liver Ratio (SLR) of approximately 0.9 [20–22]. High splenic uptake or high SLR has been observed in patients with infections as well as patients with cancers associated with an inflammatory state, such as Hodgkin’s disease [23] or cholangiocarcinoma [21, 22, 24]. There have also been reports of immune activation as a result of interferon alpha 2b or anti-CTLA4 [25]. Hence PET evaluation of immune organs of patients receiving immunotherapy may be of clinical value. In this article, we analysed baseline FDG PET/CT performed on patients prior to immunotherapy and its correlation with clinical outcome to identify novel PET imaging biomarkers.

## Methods

### Patients

All patients with unresectable stage III or stage IV melanoma (with progressive disease) who received monotherapy with ipilimumab or anti-PD1 (either pembrolizumab or nivolumab) and had completed a pre-treatment FDG PET scan at Peter MacCallum Cancer Centre (PMCC) between 1st July 2010 and 30^th^June 2015 were included. Prior treatment with BRAF targeted therapies was permissible but patients treated with BRAF/MEK inhibitors post progression on ipilimumab or anti-PD1 were excluded. Ipilimumab was given at 3 mg/kg intravenously every 3 weeks for four doses. Pembrolizumab was given at 2 mg/kg intravenously every 3 weeks, and nivolumab was given at 3 mg/kg intravenously every 2 weeks until disease progression. Patients received anti-PD1 either as monotherapy or upon progression after ipilimumab treatment (i.e. concurrent ipilimumab and nivolumab treatment was not included). Medical records were retrospectively reviewed for patient demographics, disease characteristics, blood parameters, treatment and clinical outcome. Treatment response was determined by local treating oncologist based on available clinical and imaging data.

We then sought to validate the association between SLR > 1.1 and OS in an additional cohort. Eligible patients treated at UZ Brussel, Brussels and University Hospital Zurich, Zurich were combined into a single validation cohort. Patients treated with at least one dose of ipilimumab were included and patient demographics, date of death or last contact were recorded. Pre-treatment PET scans were assessed for SLR by the local nuclear medicine expert. The final statistical analysis was performed at PMCC. This study was approved by human research ethics committees and the requirement for informed consent was waived at the PMCC, the UZ Brussel, and the University Hospital Zurich.

### FDG-PET/CT imaging analysis

**Fig. 1:**
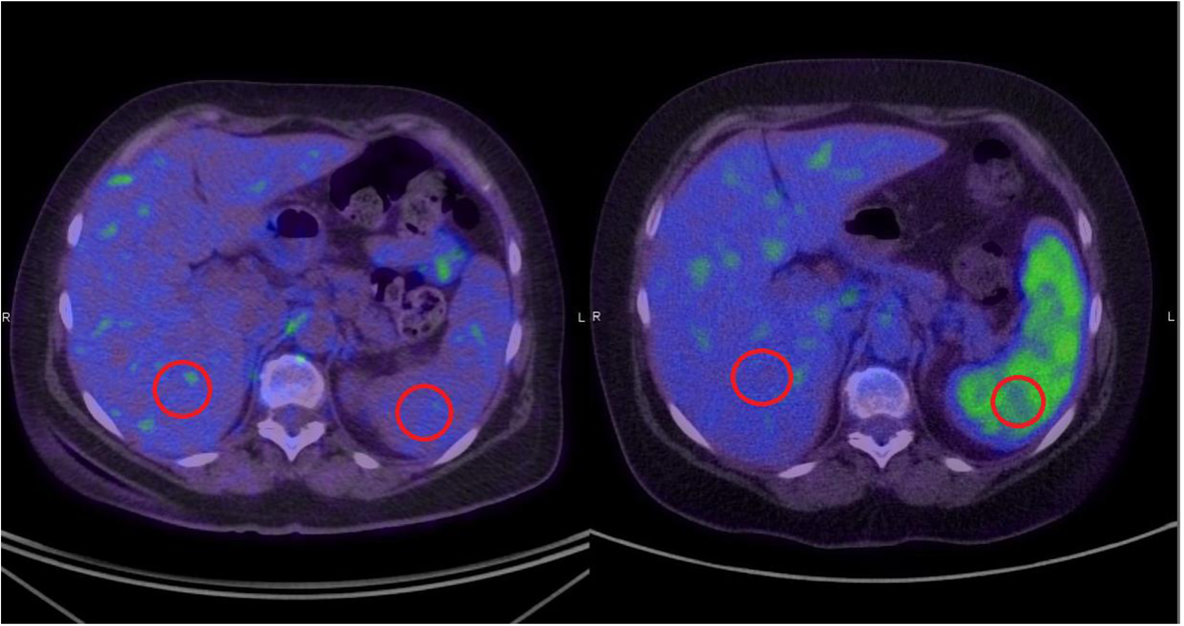
Spleen to Liver Ratio (SLR) on positron emission tomography. Example of normal splenic FDG tracer uptake (left) compared to abnormally high tracer uptake within the spleen (right). The SUV is calculated by placing 2 cm spherical volumes of interest in the liver and spleen respectively (as depicted by red circles). The spleen to liver ratio (SLR) is calculated by dividing the SUVmean of the spleen by the SUVmean of the liver. SLR > 1.1 is considered abnormally high

Pre-treatment PET scans were performed within 6 weeks before commencing ipilimumab or anti-PD1. At PMCC, the analysis was performed by using MIM software, which provided an automated volume-of-interest analysis on the basis of user-defined search regions. Parameters evaluated included the SUVmax for all tumour lesions, MTV and SLR. Metabolic tumour volume (mL), was calculated by multiplying the number of abnormal voxels identified within the volume of interest by the known voxel volume by adapting the PERCIST recommendations for contouring FDG-avid disease [26]. All FDG-avid disease was contoured using an SUV threshold, which was applied to the whole body. The SUV threshold used was 1.5 times the mean SUV of the liver plus 2 standard deviations. The SLR is calculated by placing 2 cm spherical volumes of interest in the liver and spleen (Fig. 1). The ratio of the SUVmean of the spleen to the SUVmean of the liver is then calculated. A SLR of greater than 1.1 was considered abnormal. If the patient had metastases in either the liver or the spleen, the volumes of interest would be placed in the adjacent normal organ tissue to avoid areas of metastases. The validation sites were only required to evaluate the SLR. Detailed PET acquisition methodology is included in *supplementary materials (**S1*).

### Statistical analyses

FDG PET/CT parameters (tumoral SUVmax, MTV and SLR) were tested for association with PFS and OS after ipilimumab and anti-PD1, with treatment line and metastasis substage considered as confounding variables [27, 28]. PFS and OS were tested for association with PET/CT parameters using Cox proportional hazards regression. The median tumoral SUVmax and MTV were calculated using all pre-treatment scans in the discovery cohort (*n* = 110). These median values were used to dichotomize the cohort to compare high tumoral SUVmax versus low tumoral SUVmax, as well as high MTV versus low MTV. Tumoral SUVmax and MTV were also analysed as continuous variables. The SLR normally varies within a tight range of 0.9–1.0; consequently SLR > 1.1 was considered abnormally high and used as the threshold for survival analyses. PFS was assessed and modelled separately for ipilimumab and anti-PD1 and was measured from the start of immunotherapy to the date of progression or death; patients were censored at date of last contact. Progression was assessed either clinically (if patient had died or was too unwell for imaging), or clinical imaging with either PET, CT or MRI. OS was assessed and modelled separately for a) ipilimumab, b) anti-PD1 and c) first line immunotherapy (either ipilimumab or anti-PD1, whichever was received first). OS was measured from start of treatment until death or censored at date of last contact. Other analyses for survival after first line immunotherapy considered LDH, metastasis substage (AJCC 7th Edition) [27], MTV, ALC and SLR. Statistical analyses were performed using the base package and the *survival* package (R version 3.4.2). Kaplan-Meier survival curves were used to present survival outcome for SUV and MTV as dichotomized at the median for the treatment cohort, or at the abnormal threshold of SLR > 1.1. The additional cohorts (UZB and UHZ) were used to validate the association of SLR with OS after ipilimumab.

### Biological analyses on discovery cohort

To explore the relationship between SLR and inflammatory and haematological parameters, a multi-variable linear regression model was built to explore predictor variables including: neutrophil: lymphocyte ratio, haemoglobin, LDH, ALC × 109, albumin, lymphocyte: monocyte ratio and patient performance status (*Supplementary materials**S2*).

A subset of patients in the discovery cohort were also enrolled in a separate biomarker study where their sera were stored at multiple time points. Pre-treatment samples were taken at baseline, prior to treatment at week 3 and prior to week 6. We sought to evaluate baseline and dynamic changes in the cytokine profile of patients with high SLR compared to those with normal SLR (*Supplementary materials**S3*). Stored serum samples were tested using the BD Cytometric Bead Array Enhanced Sensitivity kit for IL1β, IL2, IL4, IL6, IL10, IL12p70, IL17A, TNF, IFNγ as per the manufacturer’s protocol. Experiments were performed in duplicate. Samples were analysed using the BD FACSVerse analyser and results summarised using Graphpad Prism.

## Results

### Patient and disease characteristics for discovery cohort

Ninety-one patients received ipilimumab or anti-PD1 with a pre-treatment FDG PET scan in the study period. One patient had metabolic tumour volume of zero and was excluded from all analyses. Of the 90 evaluable patients: 50 patients received ipilimumab only, 20 patients received anti-PD1 only and 20 patients received ipilimumab followed by anti-PD1. The cohort of patients is summarised in Table 1. The median age of patients was 61 years, 67% were men, and 67% had visceral M1c disease. One patient was excluded from the analyses for SLR as she had previously undergone a splenectomy.

**Table 1:** Patient clinical and disease characteristics

Patient characteristic	***n*** = 90
**Age**	
Mean	60.6
Range	30–87
**Sex**	
Female	30 (33%)
Male	60 (67%)
**Stage**	
IIIC	5 (6%)
M1a	7 (8%)
M1b	17 (19%)
M1c	61 (67%)
**Mutation**	
BRAFV600E	16 (18%)
BRAFV600K	2 (2%)
BRAF other	5 (6%)
RAS	9 (10%)
Other	1 (1%)
No mutation	54 (60%)
Not reported	3 (3%)
**Lactate dehydrogenase (*n*** **= 78)**	
< 1xULN	26 (33%)
> 1xULN	52 (67%)
**Previous lines of therapy**	
0	52 (58%)
1	30 (33%)
2	8 (9%)

### Pre-treatment FDG PET and patient survival in discovery cohort

The 90 patients had a median tumoral SUVmax of 16.6 (Range 2.2–78.2, IQR 12.1–23.7), median MTV of 60.2 ml (Range 0.03–2693.7, IQR 13.5–212.7) and median SLR of 0.9 (Range 0.7–1.4, IQR 0.8–1.0) shown in Table 2. The baseline PET parameters of all evaluable patients in the discovery cohort (*n* = 90) are shown in Table 2. The baseline PET parameters prior to ipilimumab treatment in the discovery cohort (*n* = 70) are shown in Table 3. The baseline PET parameters prior to anti-PD1in the discovery cohort (*n* = 40) are shown in Table 4.

**Table 2:** Baseline PET parameters of discovery cohort (all patients treated with immunotherapy, *n* = 90)

PET parameter	Mean	Median	Range	IQR
**Tumoral SUV max**	20.3	16.6	[2.2, 78.21]	12.12–23.74
**SUV mean**	6.9	6.1	[1.7, 18.92]	5.33–7.54
**MTV (mL)**	197.6	60.2	[0.03, 2694]	13.47–212.74
**SLR**	0.9	0.9	[0.669, 1.364]	0.81–0.97

*PET* Positron emission tomography; *IQR* Interquarter range; *SUVmax* Maximum Standardised Uptake Value; *SUVmean* Mean Standardised Uptake Value; *MTV* Metabolic Tumour Volume; *SLR* Spleen to Liver Ratio

**Table 3:** Baseline PET parameters of discovery cohort (patients treated with ipilimumab, *n* = 70)

PET parameter	Mean	Median	Range	IQR
**Tumoral SUV max**	19.3	16.1	[2.2,71.8]	[11.6,21.5]
**SUV mean**	6.8	6.1	[1.7, 18.9]	[5.1–7.5]
**MTV (mL)**	175.2	36.3	[0.03,2693.7]	[12.5167.1]
**SLR**	0.9	0.9	[0.669, 1.364]	0.81–0.97

*PET* Positron emission tomography; *IQR* Interquarter range; *SUVmax* Maximum Standardised Uptake Value; *SUVmean* Mean Standardised Uptake Value; *MTV* Metabolic Tumour Volume; *SLR* Spleen to Liver Ratio

**Table 4:** Baseline PET parameters of discovery cohort (patients treated with antiPD1, *n* = 40)

PET parameter	Mean	Median	Range	IQR
**Tumoral SUV max**	20.3	16.6	[2.2, 78.21]	12.12–23.74
**SUV mean**	6.9	6.1	[1.7, 18.92]	5.33–7.54
**MTV (mL)**	197.6	60.2	[0.03, 2694]	13.47–212.74
**SLR**	0.9	0.9	[0.669, 1.364]	0.81–0.97

*PET* Positron emission tomography; *IQR* Interquarter range; *SUVmax* Maximum Standardised Uptake Value; *SUVmean* Mean Standardised Uptake Value; *MTV* Metabolic Tumour Volume; *SLR* Spleen to Liver Ratio

Tumoral SUVmax was not significantly associated with PFS after ipilimumab or antiPD1 when dichotomized at the median of the cohort (HR 0.78, *p* = 0.348 and HR 0.81, *p* = 0.560 respectively). See Table 5*.* Tumoral SUVmax was also not significantly associated with PFS when analysed as a continuous variable, HR 1.00 for ipilimumab with *p* = 0.74 and HR 0.98 for anti-PD1 with *p* = 0.23. MTV was not statistically significant in its association with PFS when dichotomized at the median of the cohort for ipilimumab (MTV > 41 ml, HR 1.45 with p = 0.23) or for anti-PD1 (MTV > 140 ml, HR 0.94 with *p* = 0.86). MTV was also not significantly associated with PFS when analysed as a continuous variable, HR 1.07 for ipilimumab with *p* = 0.07; and HR = 1.00 for anti-PD1 with *p* = 0.86. However, strikingly, high SLR > 1.1 was associated with poor PFS after ipilimumab compared to patients with normal SLR (median PFS 1.0 vs 3.0 months, HR 3.14, *p* = 0.008), but was not associated with PFS after anti-PD1 (median 3.0 vs 3.0 months, HR 0.56, *p* = 0.324).

**Table 5:** Association between PET parameters and progression free survival, for ipilimumab and anti-PD1 treatment, analysed as continuous variable and dichotomized at the median for the cohort

	Ipilimumab (***n*** = 70)					Anti-PD1 (***n*** = 40)				
**PET** **parameter**	**Level**	**n**	**HR**	**HR 95% CI**	***P*-value**	**Level**	**n**	**HR**	**HR 95% CI**	***P*-value**
**Tumoral SUVmax**			0.997	[0.978, 1.02]	0.74			0.984	[0.958, 1.01]	0.23
**MTV (mL)**			1.07	[1.01, 1.14]	0.066			0.993	[0.924, 1.07]	0.86
**SLR**			8.24	[1.08, 63.1]	0.05			0.904	[0.0623, 13.1]	0.94
**Tumoral SUVmax**	≤18	39	1	–	0.348	≤18	16	1	–	0.56
	> 18	31	0.78	[0.46, 1.31]		> 18	24	0.81	[0.39, 1.66]	
**MTV (mL)**	≤41	34	1	–	0.231	≤140	20	1	–	0.86
	> 41	36	1.45	[0.79, 2.68]		> 140	20	0.94	[0.46, 1.90]	
**SLR**	≤1.1	60	1	–	0.008	≤1.1	35	1	–	0.32
	> 1.1	9	3.14	[1.46, 6.75]		> 1.1	5	0.56	[0.16, 1.92]	

*Anti-PD1* Anti-programmed death 1 monoclonal antibody; *PET* Positron emission tomography; *HR* Hazard ratio; *CI* Confidence interval; *SUVmax* Maximum Standardised Uptake Value; *SUVmean* Mean Standardised Uptake Value; *MTV* Metabolic Tumour Volume

**Fig. 2:**
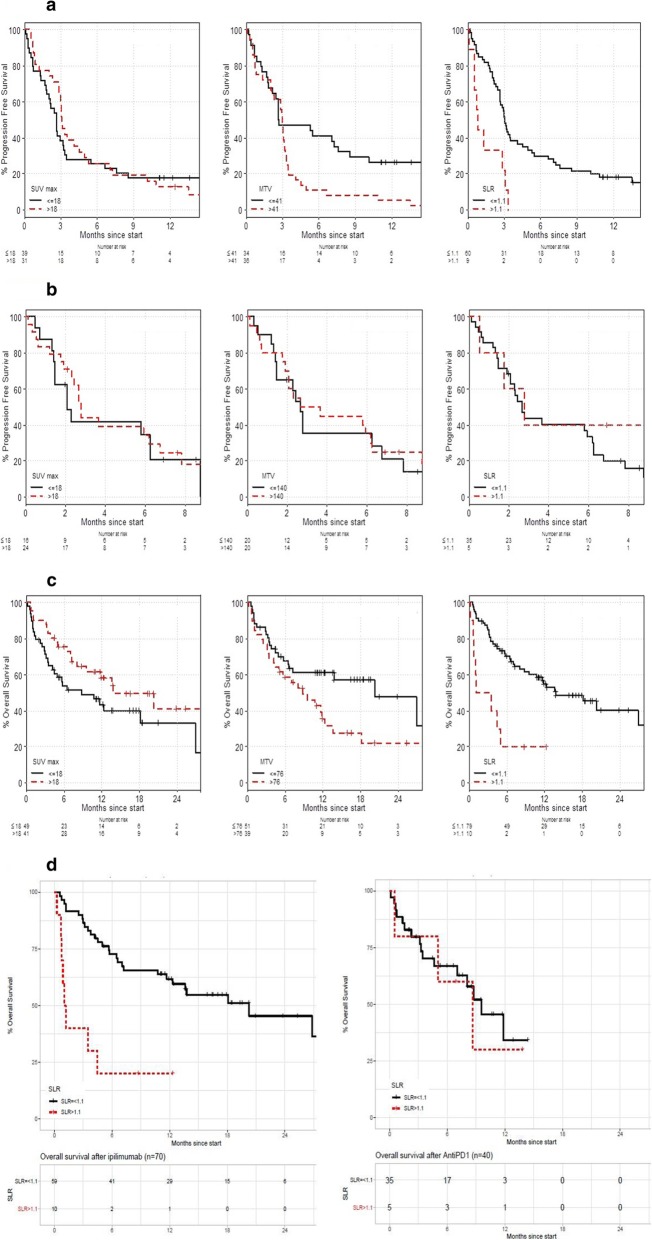
**a**. Kaplan Meier curves of FDG PET parameters and Progression free survival after ipilimumab. **b**. Kaplan Meier curves of FDG-PET parameters and Progression free survival after anti-PD1. **c**. Kaplan Meier curves of FDG-PET parameters and overall survival from the start of first line immunotherapy. **d**. Kaplan Meier curves of Spleen to Liver Ratio (SLR) and overall survival after ipilimumab and anti-PD1 respectively. (A) and (B). Kaplan Meier survival curves for progression free survival after ipilimumab or anti-PD1 and its correlation for SUVmax, Metabolic Tumour Volume (MTV) and spleen to liver ratio (SLR) respectively. High SLR was significantly correlated with PFS after ipilimumab (median PFS 1.0 vs 3.0 months, HR 3.14, *p* = 0.008), but was not associated with PFS after anti-PD1 (median 3.0 vs 3.0 months, HR 0.56, *p* = 0.324). Figure 2(c) High SLR was associated with poor OS after first line immunotherapy (ipilimumab or anti-PD1), median 1 vs 14 months; HR 3.92, *p* = 0.003. Neither SUVmax nor MTV was significantly associated with PFS or OS after either treatment. Figure 2(D) High SLR was associated with poor OS after ipilimumab (median 1 v 21 months, HR 5.83, *p* = 0.0001. High SLR was not associated with OS after anti-PD1 treatment (median 8.8 v 9.7 months; HR 0.92, *p* = 0.89)

Overall survival was calculated from date of first treatment separately for each agent, as well as for date of first line immunotherapy. High SLR was also associated with short OS as calculated from start of first line immunotherapy (median 1.0 month vs 14.0 months respectively, HR 3.92, *p* = 0.003). See Table 6 and Fig. 2c*.* Patients with high SLR > 1.1 also had significantly worse OS compared to patients with normal SLR after ipilimumab (median 1 vs 21 months; HR 5.83, *p* = 0.0001). See Fig. 2d*.* High SLR was not associated with OS after anti-PD1 treatment (median 8.8 v 9.7 months; HR 0.92, *p* = 0.89).

**Table 6:** Association between PET imaging parameters and overall survival, analysed as continuous variable and dichotomized at the median for the cohort

	Ipilimumab (***n*** = 70)				
**PET parameter**	**Level**	**n**	**HR**	**HR 95% CI**	***P*-value**
**Tumoral SUVmax**			0.983	[0.95,1.01]	0.24
**MTV**			1.000	[1.00, 1.001]	0.03
**SLR**			18.71	[1.34, 261.25]	0.02
**Tumoral SUVmax**	< 16.07	35	1	–	
	> 16.07	35	0.83	[0.42, 1.63]	0.59
**MTV (mL)**	< 41.38	35	1	–	
	> 41.38	35	1.09	[0.52, 2.29]	0.83
**SLR**	< 1.1	59	1	–	
	> 1.1	10	5.83	[2.31–14.74]	0.0002
	**Anti-PD1 (*n*** **= 40)**				
**PET parameter**	**Level**	**n**	**HR**	**HR 95% CI**	***P*-value**
**Tumoral SUVmax**			0.967	[0.93, 1.007]	0.10
**MTV**			1.000	[0.9995, 1.001]	0.49
**SLR**			2.419	[0.08, 76.00]	0.62
**Tumoral SUVmax**	< 19.33	20	1	–	
	> 19.33	20	0.57	[0.21, 1.53]	0.27
**MTV (mL)**	< 137	20	1	–	
	> 137	20	1.67	[0.64, 4.34]	0.29
**SLR**	< 1.1	35	1	–	
	> 1.1	5	0.92	[0.26, 3.29]	0.89
	**First line immunotherapy (*n*** **= 90)**				
**PET parameter**	**Level**	**n**	**HR**	**HR 95% CI**	***P*-value**
**Tumoral SUVmax**			0.986	[0.964, 1.01]	0.21
**MTV**			1.09	[1.02, 1.15]	0.02
**SLR**			17.3	[1.90, 157]	0.02
**Tumoral SUVmax**	≤18	49	1	–	0.05
	> 18	41	0.57	[0.32, 1.01]	
**MTV (mL)**	≤76	51	1	–	0.61
	> 76	39	1.18	[0.63, 2.23]	
**SLR**	≤1.1	79	1	–	0.003
	> 1.1	10	3.92	[1.76, 8.72]	

*PET* Positron emission tomography; *SUVmax* Maximum Standardised Uptake Value; *MTV* Metabolic Tumour Volume; *SLR* Spleen to Liver Ratio

Again, high tumoral SUVmax was not associated with OS after first line immunotherapy compared to low tumoral SUVmax, (median OS 14.0 vs 9.0 months, HR 0.57, *p* = 0.053). High tumoral SUVmax was not associated with OS after ipilimumab (HR 0.83, *p* = 0.59) or after anti-PD1 (HR 0.57, *p* = 0.267). SUVmax also not significantly associated with OS after first line immunotherapy when analysed as a continuous variable, HR 0.99 with *p* = 0.21.

**Fig. 3:**
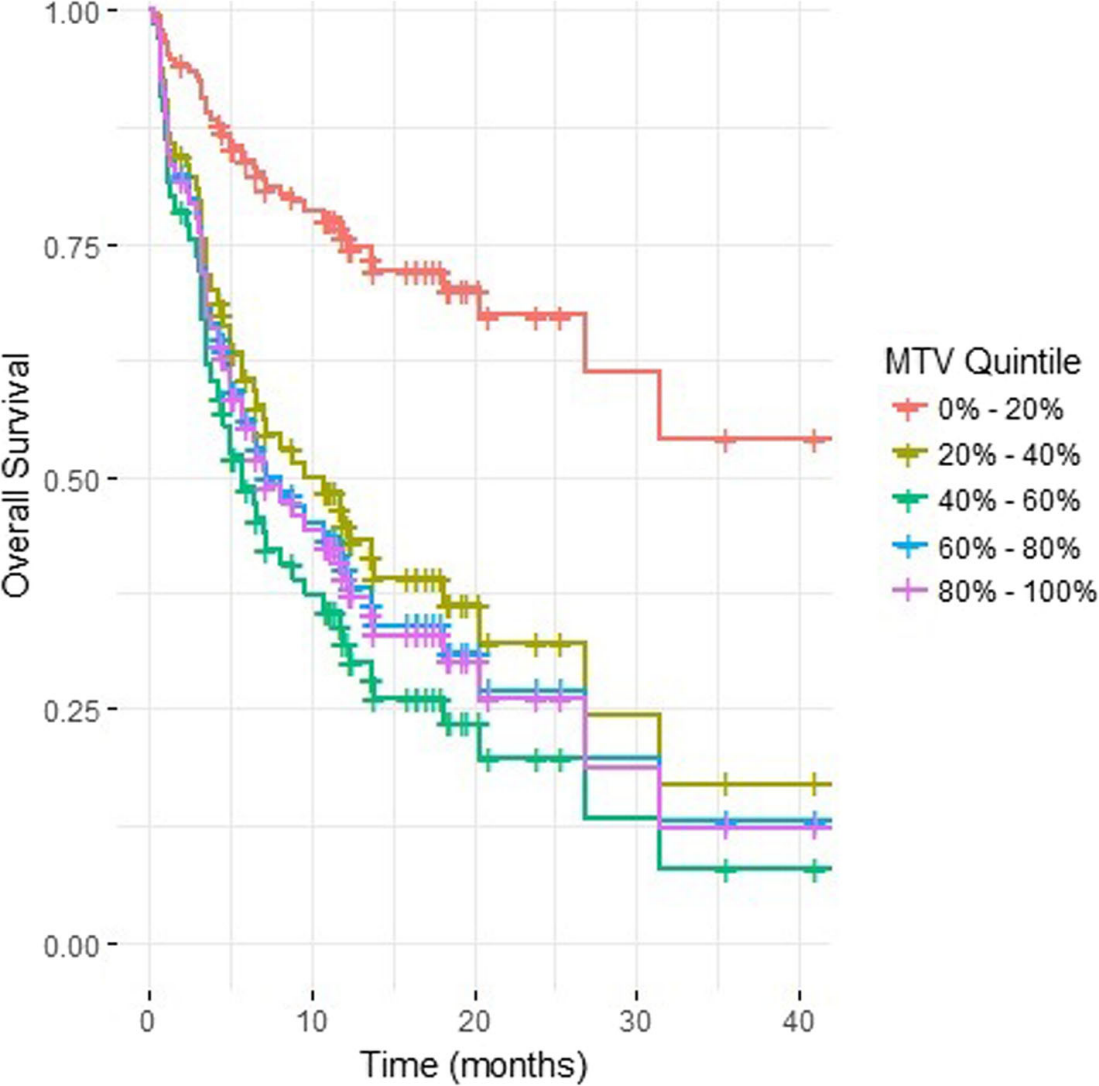
Overall survival curves stratified by metabolic tumour volume quintile. High metabolic tumour volume (MTV) was not associated with OS compared to patients with low MTV when dichotomised at the median (HR 1.18, *p* = 0.606) but MTV was significantly associated with OS when analysed as a continuous variable (HR 1.09, *p* = 0.020). To explore this further, the discovery cohort was split into MTV quintiles and found that the quintile with the lowest level of disease had excellent OS whereas the remaining 4 quintiles all appeared to have similar OS. This suggests a degree of non-linearity between the MTV and OS

Interestingly, high MTV were not associated with OS after first line immunotherapy compared to patients with low MTV when dichotomised at the median (median OS 9.0 vs 20.0 months, HR 1.18, *p* = 0.606) but MTV was significantly associated with OS when analysed as a continuous variable (HR 1.09, *p* = 0.020). Similarly, high MTV was not associated with OS after ipilimumab (HR 1.08, *p* = 0.827) or after anti-PD1 (HR 1.67, *p* = 0.294). We explored this further by splitting the cohort into MTV quintiles and found that the quintile with the lowest level of disease had excellent OS whereas the remaining 4 quintiles all appeared to have similar OS. This suggests a degree of non-linearity between the MTV and OS with excellent survival in patients with very low volume of disease as shown in Fig. 3*.*

### Multivariable analysis for progression free survival and overall survival in discovery cohort

On multivariable analysis, SLR was independently associated with survival, with HR 3.58 (95% CI 1.34–9.61), p 0.0026. The other variables including LDH, metastasis substage, MTV, absolute lymphocyte count (ALC) were all non-significant*.* See Tables 7 and 8*.*

**Table 7:** Multivariate analysis for progression free survival after ipilimumab

Predictor	Level	HR	HR 95% CI	***P***-value
**LDH**	≤ULN	1	–	0.738
	>ULN	1.12	[0.58, 2.14]	
**Stage**	not M1c	1	–	0.431
	M1c	1.32	[0.66, 2.67]	
**MTV**	≤41	1	–	0.334
	> 41	1.39	[0.71, 2.70]	
**ALC**	≤1	1	–	0.083
	> 1	0.55	[0.28, 1.06]	
**SLR**	≤1.1	1	–	0.042
	> 1.1	2.67	[1.13, 6.33]	

*LDH* Lactate dehydrogenase; *ULN* Upper limit of normal; *MTV* Metabolic Tumour Volume; *ALC* Absolute Lymphocyte Count; *SLR* Spleen to Liver Ratio

**Table 8:** Multivariate analysis for overall survival after first line immunotherapy

Predictor	Level	HR	HR 95% CI	***P***-value
**LDH**	≤ULN	1	–	0.076
	>ULN	2.13	[0.89, 5.08]	
**Stage**	not M1c	1	–	0.163
	M1c	1.88	[0.75, 4.75]	
**MTV**	≤76	1	–	0.973
	> 76	1.01	[0.51, 2.02]	
**ALC**	≤1	1	–	0.164
	> 1	0.61	[0.30, 1.21]	
**SLR**	≤1.1	1	–	0.026
	> 1.1	3.58	[1.34, 9.61]	

*LDH* Lactate dehydrogenase; *ULN* Upper limit of normal; *MTV* Metabolic Tumour Volume; *ALC* Absolute Lymphocyte Count; *SLR* Spleen to Liver Ratio

For PFS analysis, multivariable analysis was performed only for the ipilimumab-treated cohort of patients, as the univariable regressions for anti-PD1 all resulted in non-statistically significant *p*-values (> 0.05). For PFS after ipilimumab, SLR was associated with HR 2.67 (95% CI 1.13–6.33), *p* = 0.042.

### Validation cohort

**Fig. 4:**
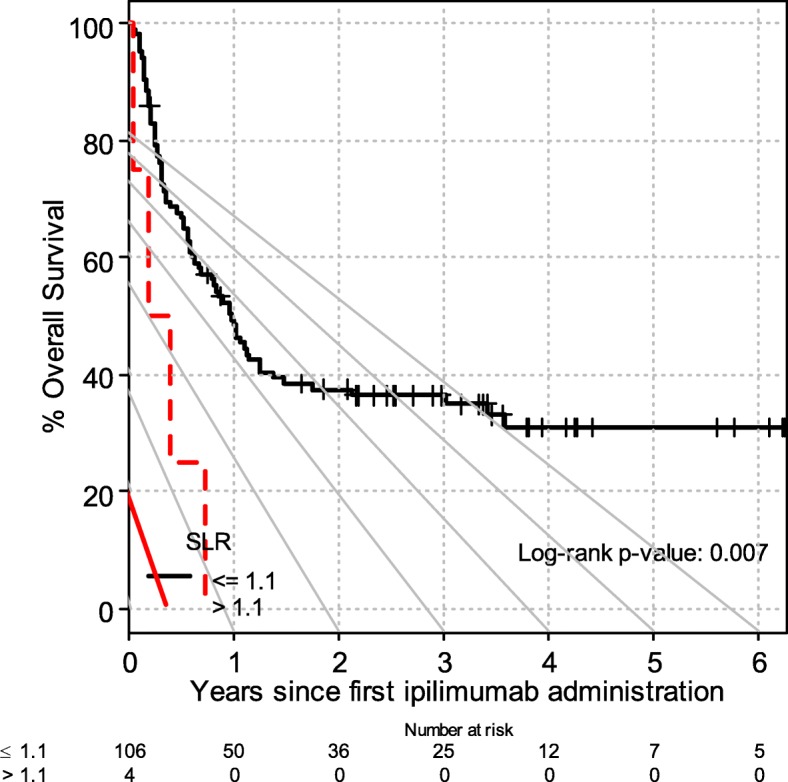
SLR and overall survival after ipilimumab in the validation cohort. High SLR was associated with poor overall survival after ipilimumab treatment. This was validated in an external cohort (*n* = 110 patients) from Brussels and Zurich (median 2.3 months versus 11.9 months, HR 3.74, CI 1.34–10.4)

One hundred ten patients treated with ipilimumab between May 2007 and April 2015 were included. Fifty-eight patients were from UZ Brussel, Brussels and 52 patients were from University Hospital Zurich, Zurich. Patient and disease characteristics are described in *Supplementary materials* (*S4)*. There were higher proportions of patients with M1c in the Brussels cohort, but this difference was negligible when the patients were combined into a single validation cohort (68% of the discovery cohort had M1c disease compared to 73% of the validation cohort). The proportion of patients with the high SLR was also lower in the European cohort compared to Australian patients (4% compared to 13%). Despite these differences, SLR > 1.1 was still associated with poor OS after ipilimumab (median 2.3 months versus 11.9 months, HR 3.74, CI 1.34–10.4), as shown in Fig. 4.

### Biological analyses for discovery cohort

We sought to explore the cause of the raised SLR. We compared the PET parameters of liver and splenic SUV in patients with raised SLR and normal SLR. In patients with raised SLR, the mean and median liver SUV was 2.0 with an interquartile range (IQR) of 0.5, which is slightly lower than the normal reference range (lower limit of normal for liver SUV was 2.3) [20]. In contrast, the normal SLR group had a mean and median liver SUV of 2.4, IQR 0.6; which is within the normal reference range. Within the high SLR group, the mean and median splenic SUV was 2.4, with IQR of 0.4; whereas in normal SLR group, where the mean and median splenic SUV was 2.1, IQR 0.5.

To explore the relationship of SLR and inflammatory or haematological parameters, a multivariable linear regression model for SLR was considered, considering: Neutrophil: Lymphocyte Ratio, Absolute Neutrophil Count (< 7.5 vs. ≥7.5), Haemoglobin (< 120 vs. ≥120), LDH (≤ULN vs. >ULN), ALC × 10^9^ (< 1 vs. ≥1), albumin (< 35 vs. ≥35), Lymphocyte: Monocyte ratio and patient performance status. Only albumin was found to be significantly associated with SLR, beta − 0.15 (CI -0.209, − 0.091, *p* < 0.001). *See supplementary materials**S2*. Given that albumin is a negative acute phase marker (i.e. decrease during inflammation), the inflammatory cytokine profile was examined in patients with stored sera.

Fourteen patients in the discovery cohort had evaluable stored sera from baseline, week 3 and week 6 post commencement of ipilimumab. Of these patients, 13 had normal SLR, whereas one patient had high SLR > 1.1. There were no major differences in the baseline levels nor the dynamic changes in IL2, IL1 beta, IL4, IL6, IL10, IL17, IL12p70, IFN gamma and TNF alpha post ipilimumab, when comparing the patient with high SLR to others with normal SLR. Patients with progressive disease and high SLR were both observed to have a logarithmic increase in IL6 and IL10 at week 3 and week 6. *See supplementary materials S3.*

## Discussion

High SLR on pre-treatment FDG-PET was associated with poor OS after ipilimumab for advanced melanoma in our study. This finding was then validated in an additional combined cohort of patients from two separate institutions. Although only a small proportion of patients exhibited high SLR (i.e. only 10% of the discovery cohort and 4% of the validation cohort), it is noteworthy that these patients had a very poor prognosis after ipilimumab (OS 1–3 months compared to 10–15 months in phase III studies) [1, 29, 30]. In the discovery cohort, high SLR was associated with poor PFS after ipilimumab but not after anti-PD1. This may be due to the small sample size of the anti-PD1 cohort or it may be due to the different mechanisms of action between the two treatments.

Caution is advised when interpreting the overall survival analyses for each immunotherapy separately. Patients in this study was treated during a transitional period where ipilimumab was the standard first line immunotherapy at the start of the study period (*n* = 50). Patients who received first line ipilimumab were able to receive anti-PD1 upon disease progression (*n* = 20). Towards the end of the study period, patients were able to receive anti-PD1 as their first line therapy (*n* = 20). The overall survival analysis of a cancer treatment could be influenced by subsequent treatments. In the case of immunotherapy, there is also the potential that overall survival is influenced by *prior* treatments given the long half-life of these agents and possible changes to the patients’ immune system following first line treatment. We attempted to address this in the study by analysing the overall survival from the start of the first line immunotherapy treatment so that patients contributed only once to survival censoring (*n* = 70). Using this analysis method, high SLR remained associated with poor OS after first line immunotherapy.

The mechanism for high splenic avidity is not well understood. The spleen, being the largest lymphoid organ in the human body, is the site of immune cell activation and maturation. Increased splenic avidity on FDG-PET has been previously reported in lymphoma, granulomatous diseases, GCSF injections and interferon-alpha administration in melanoma [31]. Pak and colleagues have also studied this phenomenon more extensively in the context of cholangiocarcinoma [21]. They showed that high splenic avidity is associated with poor survival in patients with metastatic cholangiocarcinoma [22]. However, this signature has not been studied prior to immune modulation with ipilimumab or anti-PD1 in the setting of advanced melanoma.

Pak et al. demonstrated that high SLR in patients with cholangiocarcinoma is associated with markers of inflammation including leucocytosis, raised CRP, raised levels of IL-1b, IL-1RA, IL-4, IL-6, IL-7 and IL-13 [21]. These inflammatory cytokines are suggestive of an acute phase inflammatory response or activation of humoral immunity. In our cohort, we found that patients with high SLR post ipilimumab were more likely to have low albumin, but importantly independent of leucocytosis or high neutrophil: lymphocyte ratio. Our cohort of 14 patients with stored sera was too small to perform comparative statistics but we did not find any striking differences in the cytokine profile of the patient with high SLR. It would be of interest to explore in future studies whether this splenic avidity is a result of systemic cytokines or preferential glucose metabolism in specific immune cells within the spleen. We postulate that the failure to control disease despite already invoking immune activation may underlie the lack of benefit from further stimulation by immunotherapy.

Additionally, the abnormal SLR group was associated with decreased hepatic uptake as well as increased splenic uptake. This raises the possibility that SLR could be related to both reduced liver SUV as well as high splenic SUV rather than either factor alone. However, our study only included a small number of patients with raised SLR signature so it would be important to explore this in larger cohorts.

No evidence of association between high tumoral SUVmax and outcome after immunotherapy was found. This may be because tumoral glucose uptake is not only due to malignant cells, but also is caused by immune cells as part of immune activation. Therefore, examination of tumoral SUVmax alone may be problematic in the context of immunotherapy and diseases in which immune modulation is important for disease outcomes. MTV was not associated with PFS in either ipilimumab or anti-PD1 in our cohort but was significantly associated with OS when analysed as a continuous variable. The patient group with very low levels of tumour volume appeared to have very good survival whereas the relationship did not appear linear at larger tumour volumes, this finding needs to be taken with caution given it has not been externally validated. However, it does appear to be in keeping with the findings of other investigators who have also correlated anti-PD1 response with tumoral burden as measured by CT [32, 33]. Our cohort predominantly evaluated patients treated with ipilimumab, and only included 40 patients treated with anti-PD1. It may be that MTV is of higher predictive value for anti-PD1 alone and should be evaluated in a larger cohort to better understand an optimal threshold for analysis, and its utility for that treatment.

From a technical standpoint, SLR may be an easier parameter to apply clinically than absolute thresholds of significance for parameters such as tumoral SUV or MTV. Despite effort to harmonise its measurement [16], tumoral SUVmax can be a more challenging parameter to reproduce in multi-centre studies, owing to the need for the same imaging systems, acquisition parameters, reconstruction parameters, and tracer uptake times in order to obtain an accurate and reproducible measurement of SUVmax. In the case of the SLR, the precise measure of SUV is less important. Examination of the same patient on multiple scanners, may produce different SUV but the ratio will remain the same. Consequently, this makes SLR a more robust measure for comparison between different study sites.

Our study does shift the focus of functional imaging of the tumour alone to the role of functional imaging of the host immune system. This additional use of PET scans gives a snapshot of the host immune status and may complement other biomarkers as suggested by Blank et al. [11]. Further understanding of the biological significance of avidity and activation of splenic avidity or draining lymphatics of the tumour may improve the understanding of not only the mechanism of action of immunotherapies, but also possible targets for future research into specific body sites contributing to treatment resistance.

Our study was limited by its retrospective nature, its relatively small sample size and lack of homogenous assessment modalities for PFS. Our findings were validated in an independent cohort albeit only in a small number of patients exhibited high SLR. The combined validation cohort had a similar proportion of patients with M1c disease but there was variation in the prior treatments received. Despite the low occurrence of high SLR, this parameter was associated with very poor clinical outcome and may be useful in identifying patients who should not receive ipilimumab treatment. This parameter also needs to be prospectively assessed in larger cohorts of patients treated with second line ipilimumab or ipilimumab combinations (ipilimumab/nivolumab).

## Conclusion

Pre-treatment high SLR on FDG PET/CT was associated with poor outcome after ipilimumab in advanced melanoma. Further prospective validation of this FDG PET/CT signature is required, particularly in larger cohorts of patients treated with anti-PD1 or ipilimumab combination therapies.

## Supplementary information

**Additional file 1 S1.** Detailed PET acquisition methodology. **S2.** Multi-variable linear regression model for spleen to liver ratio. **S3.** Cytokine profile of patients treated with ipilimumab (*n* = 14). **S4.** Baseline demographic and disease characteristics of validation cohort.

## Data Availability

The datasets generated and/or analysed during the current study are not publicly available due to restrictions on patients’ consent pertaining to existing ethics-committee approved research at our respective institutions only. They may be obtained from the corresponding author on reasonable request but may require further approval from respective ethics committee approval.

## Abbreviations

ALC: Absolute lymphocyte count
Anti-PD1: Anti-programmed death 1 monoclonal antibodies
BLR: Bone to Liver Ratio
FDG PET: Fluorodeoxyglucose positron emission tomography
HR: Hazard ratio
IQR: Interquartile range
LDH: Lactate dehydrogenase
MTV: Metabolic tumour volume
OS: Overall survival
PET: Positron emission tomography
PFS: Progression free survival
PMCC: Peter MacCallum cancer centre
SLR: Spleen to liver ratio
SUVmax: Maximum standardised uptake value
ULN: Upper limit of normal
