# Supplementary material for: 18F-FDG PET/CT based spleen to liver ratio associates with clinical outcome to ipilimumab in patients with metastatic melanoma
Source: Cancer Imaging. 2020 May 14;20:36. doi: 10.1186/s40644-020-00313-2 (PMC7227105; doi:10.1186/s40644-020-00313-2)
Supplement: Supplementary file 1 — Additional file 1 S1. Detailed PET acquisition methodology. S2. Multi-variable linear regression model for spleen to liver ratio. S3. Cytokine profile of patients treated with ipilimumab (n = 14). S4. Baseline demographic and disease characteristics of validation cohort. [file 40644_2020_313_MOESM1_ESM.docx]

**Supplementary Material**

S1. Detailed PET Analysis protocol

FDG-PET scans were performed after patients fasted for at least 6 hours, and plasma glucose levels were determined to document euglycemia. 18FDG was administered intravenously according to weight (0.15 mCi/kg, ranging from 3 to 16 mCi) and images were acquired after a distribution time of approximately 60 minutes. Images were obtained from the base of the skull to extremities. FDG-PET images were corrected for attenuation. Metabolic tumor volume was calculated by multiplying the number of abnormal voxels identified within the volume of interest by the known voxel volume.

The Spleen to liver ratio is calculated by placing spherical volumes of interest that are 2cm in diameter in the Liver and spleen. The ratio of the SUVmean of the spleen to the SUVmean of the liver is then calculated. A spleen to liver ratio of greater than 1.1 is considered abnormal. Below is an example of normal (left) and abnormal spleen to liver ratios (right).


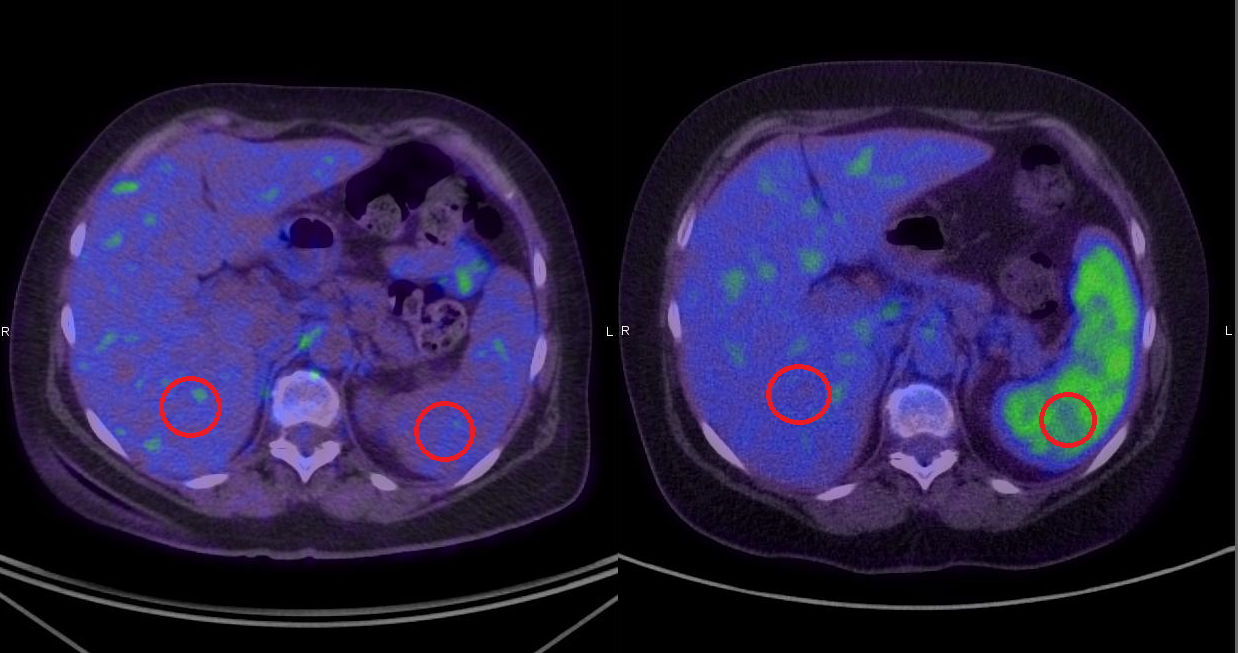


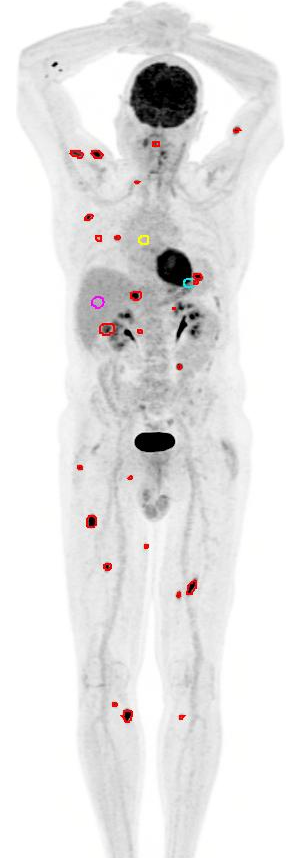
The whole body metabolic tumour volume (MTV), measured in mL, is quantified on the FDG-PET/CT scans by adapting the PERCIST recommendations for contouring FDG avid disease (R. L. Wahl, H. Jacene, Y. Kasamon and M. A. Lodge, J Nucl Med 50 Suppl 1, 122S-150S (2009)). All FDG-avid disease was contoured using an SUV threshold that was applied to the whole body.  The SUV threshold used was 1.5 times the mean SUV of the liver plus 2 standard deviations.  All normal physiologic uptake (brain, kidneys, bladder etc) was manually excluded from the whole body volume of interest as shown in the example below. The following parameters for the Whole Body disease volume of interest are recorded:

- SUVmax
- SUVmean
- Metabolic Tumour Volume (ml)

S2. Multivariate analysis for spleen to liver ratio

A multivariate linear regression model for SLR was considered, with all of the following considered as candidate covariates: NLR, Neutrophils (<7.5 vs. ≥7.5), Hb (<120 vs. ≥120), LDH (≤ULN vs. >ULN), ALC x109 (<1 vs. ≥1), Albumin (<35 vs. ≥35), LMR and ECOG-PS. (NLR = Neutrophil to Lymphocyte ratio, LMR = Lymphocyte to Monocyte ratio). A forward selection approach to model construction was used, whereby, commencing with the null model, the most significant covariate not in the model was added successively until no further covariates significant at the 0.05 level could be found. Using this approach only 1 covariate from the list above was found to be significant. Beta is the modelled slope of the regression line. In this case, since the predictor (Albumin) is dichotomous, it represents the modelled difference in SLR between patients with albumin <35 and patients with albumin ≥35.

*Multivariate linear regression results for SLR*

| Predictor | Level | beta | beta 95% CI | N | P-value |
| --- | --- | --- | --- | --- | --- |
| Albumin | <35 | 0 | - | 21 | <0.001 |
|  | ≥35 | -0.15 | [-0.209, -0.091] | 67 |  |

S3. Dynamic changes of cytokine levels in patients treated with Ipilimumab at baseline and 6 weeks post treatment (n=14).

See additional PDF

S4. Baseline demographic and disease characteristics of validation cohort

| Demographic or Characteristic | | Site | |
| --- | --- | --- | --- |
|  |  | Brussels (n = 58) | Zurich (n = 52) |
| Sex | Female | 28 (48%) | 26 (50%) |
|  | Male | 30 (52%) | 26 (50%) |
| Stage | IIIC | 2 (3%) | 5 (10%) |
|  | M1a | 4 (7%) | 4 (8%) |
|  | M1b | 3 (5%) | 11 (21%) |
|  | M1c | 49 (84%) | 32 (62%) |
| Mutation | BRAF V600E/K | 16 (28%) | 7 (13%) |
|  | RAS/NRAS | 0 (0%) | 16 (31%) |
|  | BRAF Wildtype | 22 (38%) | 25 (48%) |
|  | Other | 2 (3%) | 2 (4%) |
|  | Unknown | 16 (28%) | 2 (4%) |
| Prior lines of therapy | 0 | 10 (17%) | 25 (48%) |
|  | 1 | 44 (76%) | 23 (44%) |
|  | 2 | 4 (7%) | 4 (8%) |
| SLR | Mean (SD) | 0.82 (0.1) | 0.90 (0.1) |
|  | Median [range] | 0.81 [0.44 - 1.28] | 0.9 [0.7 - 1.3] |
|  | IQR | 0.73 - 0.89 | 0.8 - 1 |
| SLR | ≤1.1 | 57 (98%) | 49 (94%) |
|  | >1.1 | 1 (2%) | 3 (6%) |
